# Organizational Climate and Teachers’ Morale: Developing a Specific Tool for the School Context – A Research Project in Italy

**DOI:** 10.3389/fpsyg.2019.02132

**Published:** 2019-09-20

**Authors:** Daniela Converso, Michela Cortini, Gloria Guidetti, Giorgia Molinengo, Ilaria Sottimano, Sara Viotti, Barbara Loera

**Affiliations:** ^1^Department of Psychology, University of Turin, Turin, Italy; ^2^Department of Psychological, Humanistic and Territorial Sciences, D’Annunzio University of Chieti–Pescara, Chieti, Italy

**Keywords:** school climate, teachers’ morale, teachers’ wellbeing, confirmatory factor analysis, adaptation, validation

## Abstract

The school context is exposed to several demanding factors relating to student and family needs and external evaluative processes of students’ learning and process outcomes, such as abilities in planning training courses and a learning environment. However, there is a need to develop tools that adequately support schools in making self-assessment evaluations of the internal organizational climate and teacher morale (TM). The present study proposes an Italian version of the School Organizational Health Questionnaire (SOHQ), developed by Hart et al. (2000). An Italian version of the SOHQ was deployed to 9 public primary schools in the north of Italy, and 325 cases were eventually retained as being valid for the analysis. Using confirmatory factor analysis, results highlight that a 56-item version is model fit and presents satisfactory psychometric properties, demonstrating the suitability of a latent structure composed of 12 intercorrelated factors. The present study gives further insight into increasing the use of self-assessment tools in the development of good practices and the monitoring of teacher morale within the school context.

## Introduction

In recent years, schools have been required to adapt and meet new demands coming from society, as well as from students and their parents. Coherently, teachers’ activities and roles become more differentiated, frequently updated, and “sophisticated,” especially in the management of their interaction with students.

Therefore, research on teachers’ wellbeing has largely focused on the conditions of stress and burnout related to organizational conditions as well as to the relationships with colleagues and also those with students and their families (Kyriacou, 2001; Cordeiro Castro et al., 2002; Salanova et al., 2005; Chaplain, 2008; Favretto and Rappagliosi, 2009; Avanzi et al., 2012; Velasco et al., 2013; Capone and Petrillo, 2016). However, the excessive relevance paid to the negative aspects of the teaching work and the consequences in terms of psychological distress has mostly neglected the role of positive aspects that could influence wellbeing. Indeed, as outlined by Hart et al. (1995), and according to the job demands-resources model (Demerouti et al., 2001; Hakanen et al., 2006), positive working experiences may exert independent roles on wellbeing outcomes (Martini et al., 2015), emphasizing the link between organizational climate and teachers’ health (e.g., Wearing et al., 1990; Hakanen et al., 2006; Skaalvik and Skaalvik, 2011; Benevene and Fiorilli, 2015; De Stasio et al., 2017).

Research has also shown how teacher wellbeing could be an important determinant for student learning outcomes and wellbeing, highlighting the importance of establishing not only the determinants of teachers’ negative outcomes, but also those of the positive ones associated with their work (Rowe, 1992; Denny et al., 2011; Maxwell et al., 2017; Benevene et al., 2018; Guidetti et al., 2018; Lei et al., 2018). Moreover, as highlighted by the health promoting schools perspective, an extension of the Ottawa Charter holistic definition of health promotion (World Health Organization [WHO], 1986), teachers’ wellbeing is part of a wider process able to sustain, at the same time, the health of children, adolescents, and the entire school community (Moon, 1999; Mitchell and Sackney, 2000; Konu and Rimpelä, 2002).

Among the factors that could be identified as achieving positive occupational wellbeing outcomes, such as job satisfaction, engagement, or commitment, the concept of morale is one of the least studied. This concept refers to the presence of energy, persistence, cohesion, and cooperation, that reflects a positive psychological state of mind (Hart et al., 2000) and can represent an important indicator of individual and group wellbeing (Peterson et al., 2008). Concerning the school context, teacher morale (TM) should be regarded as the professional interest and enthusiasm displayed toward the achievement of individual and group goals within the school setting (Bentley and Rempel, 1980). Therefore, it represents a form of a positive mental and emotional state that has the power to establish the character of a school. In addition, TM is considered one of the leading factors in determining the best functionality of a school (Eboka, 2017). In spite of this, past studies developed a measure of TM that was referred to as “a number of heterogeneous items related to adjustment and mental health” (Doherty, 1988, p. 72) that are more likely to derive dispositional levels of negative effects, instead of positive ones. Hart and Conn (1992) stated, on the contrary, that morale is a construct associated with enthusiasm, energy, and group spirit that teachers feel as a unique experience at work. Moreover, as stated by Hart et al. (2000), measurement tools, like the staff morale questionnaire (Meaney and Smith, 1988), failed to differentiate the causes from the manifestations of morale. According to Evans (1992, p. 832), TM could be defined as “a state of mind determined by the individual’s anticipation of the extent of satisfaction of those needs which s/he perceives as significantly affecting her/his total work situation.” Based on this definition, TM is thus intended to evaluate a future-oriented perspective, and in line with this, it is of upmost importance to identify the conditions under which TM could be fed.

In order to understand factors that could affect TM, the School Organizational Health Questionnaire (SOHQ) was developed by Hart et al. (2000) in Australia, and mainly adopted in English speaking countries (Stewart et al., 2004; Burns and Machin, 2012) and China (Wang, 2009; Wang et al., 2013). Recognizing the elements that are typically addressed by organizational development processes, the questionnaire was intended to measure different aspects that characterize a healthy organizational school climate, moving on from the assumption that organizational factors are much more important than specific classroom factors in influencing TM. Organizational climate has been defined as a multidimensional construct that refers to a variety of individual evaluations of the work environment (James and James, 1989; Neal et al., 2000). Therefore, it is based on how individuals attribute meaning to their organizational environment, and, in the school context, may concern the interpersonal relationships, such as staff affiliation and student support and organizational behavior and aspects of human resource management, such as appraisal and recognition, professional growth, and role clarity. Identifying factors of a healthy school environment thus serves in taking into account the complexity of a school as a social system where different roles – administrative, teaching, and learning – constantly interact, and in monitoring the school’s effectiveness in performing its various functions (Tsui and Cheng, 1999).

The SOHQ (Hart et al., 2000) has then been developed in order to evaluate a healthy school environment, tackling some limitations of past instruments [see, for example, the School Level Environment Questionnaire (SLEQ, Rentoul and Fraser, 1983); the Organizational Climate Description Questionnaire (OCDQ, Haplin and Croft, 1963); or the Organizational Health Inventory (OHI, Hoy and Feldman, 1987)]: the restricted focus on social interaction between teachers and the principal proposed by the OCDQ (Haplin and Croft, 1963) and the absence of human resource management characteristics within the OHI (Hoy and Feldman, 1987) and the SLEQ (Rentoul and Fraser, 1983), such as appraisal and recognition, opportunities for professional growth, and role clarity, which past studies have shown to be central elements for teachers’ occupational wellbeing (Kelchtermans and Strittmatter, 1999; Kokkinos, 2007).

Therefore, the SOHQ (Hart et al., 2000) has been developed with the aim of providing a broader emphasis on aspects concerning organizational behavior and human resource management within schools and, differently from the other tools, it could represent a more tailored, context-specific questionnaire for assessing teachers’ needs. The eleven organizational dimensions that authors highlighted, after their preliminary studies, for defining a healthy organizational school climate are the following: *Appraisal & Recognition* (having feedback, being encouraged), *Curriculum Coordination* (interprofessional collaboration and contacts), *Effective Discipline Policy* (agreement on discipline and rules between teachers), *Excessive Work Demands* (workload perception), *Goal Congruence* (commitment, clear and agreed values and aims), *Participative Decision Making* (perception of being included in school policies), *Professional Growth* (satisfaction of personal and professional development in the school), *Professional Interaction* (social support and positive interpersonal climate), *Role Clarity* (having clear role expectations), *Student Orientation* (positive climate toward students), and *Support Leadership* (quality of school leadership).

The present study aims to propose an adaptation and validation of the SOHQ (Hart et al., 2000) for the Italian context and a tool for assessing systematically different factors related to the school climate and teachers’ wellbeing and for better understanding criticisms and resources that can reduce or strengthen students’ wellbeing and learning outcomes.

## Materials and Methods

### Data Collection and Ethics Statement

Data were collected through a survey, using a cross-sectional and non-randomized design. Teachers from nine public primary schools in a region of Northern Italy were involved in this survey. The study database originally included 407 participants (22% of the entire population), but 82 of them avoided responding to some items. The final dataset used in the analysis consisted of 325 valid cases. According to the target population specificity, most participants were female (93.8%), with a mean age of 44.7 (SD = 9.7) and an average length of experience of 20 years (SD = 11.2). Moreover, 70.6% of the sample was composed of married persons with children, and 17.8% had at least one adult relative to care for. Generally, participants declared that they were quite satisfied with their work and appreciated their life in general: the mean judgments were equal to 7.9 and 8.2, respectively, on a scale of ten points.

Data were gathered using self-report questionnaires distributed to all employed teachers during working hours. To ensure anonymity, teachers were instructed to enclose the completed questionnaire in an envelope and leave it in a box placed by the researchers in each school. The participants volunteered for the research without receiving any reward and agreed to anonymously complete the questionnaire, signing the informed consent forms, which were placed in a box before completing the questionnaire. The research conforms to the Declaration of Helsinki of 1995 (as revised in Edinburgh, 2000), and all ethical guidelines were followed, as required for conducting human research, including adherence to the legal requirements of the country under study. Additional ethical approval was not required because no treatment was involved, including medical, invasive diagnostics, or procedures causing psychological or social discomfort for the participants.

### Measures

The self-reported questionnaire included a sociodemographic section and the SOHQ (Hart et al., 2000). The original version of the SOHQ consisted of 57 Likert-type items (from 1 = completely disagree to 4 = completely agree), grouped in 12 sub-dimensions: Morale (M, 5 items), Appraisal & Recognition (AR, 6 items), Curriculum Coordination (CC, 4 items), Effective Discipline Policy (EDP, 4 items), Excessive Work Demands (EWD, 4 items), Goal Congruence (GC, 5 items), Participative Decision Making (PDM, 4 items), Professional Growth (PG, 5 items), Professional Interaction (PI, 7 items), Role Clarity (RC, 4 items), Student Orientation (SO, 4 items), and Support Leadership (SL, 4 items). All items included in the version presented in the study by Hart et al. (2000) were translated from English into Italian, following the International Guideline on Test Adaptation (International Test Commission [ITC], 2015).

On the basis of previous literature (Dolbier et al., 2005; Kristensen et al., 2005), *job satisfaction* and *general life satisfaction* were measured by a single item each [i.e., “Taking everything into consideration, how satisfied do you feel with your job (or life) as a whole?”] Each item was rated on a 10-point scale that ranged between 1 (extremely dissatisfied) and 10 (extremely satisfied).

### Statistical Analyses

The SOHQ items were preliminarily analyzed with descriptive statistics; Cronbach’s alpha coefficient was calculated in order to assess scale reliability whereas the contribution of each item to internal consistency was examined by the item–total correlations.

SOHQ dimensionality was investigated by confirmatory factor analysis (CFA), which is recommended over exploratory factor analysis (EFA) because it allows testing of whether the data fit a structure when there is an *a priori* hypothesis regarding dimensionality (Floyd and Widaman, 1995); the SOHQ questionnaire was designed to measure TM and 11 dimensions of school organizational climate which were both theoretically and empirically distinct.

The model fit was considered acceptable if the following criteria were satisfied: root mean square error of approximation (RMSEA) <0.08; comparative fit index (CFI) >0.90; and standardized root mean square residual (SRMR) <0.08 (Hu and Bentler, 1995, 1999).

The analyses were performed using the IBM SPSS Statistics 25.0 and Mplus7 software programs.

## Results

Descriptive statistics for the items are shown in Table 1. For all items, the corrected item–total correlation achieved values equal or greater than *r* = 0.40, except for item 45 that reported a value of 0.29. All skewness and kurtosis values are included in the range from –1.0 to +1.0.

**TABLE 1:** Items distribution: descriptive, and reliability analysis by subdimension.

	**Code**	**M**	**Sd**	**S**	**K**	**Corr. item–total**
Morale (α_tot_ = 0.85)	B1	2.99	0.683	–0.499	0.636	0.560
	B3	2.97	0.702	–0.436	0.331	0.754
	B4	2.70	0.724	–0.286	–0.043	0.606
	B5	2.78	0.786	–0.331	–0.205	0.731
	B12	2.59	0.731	–0.398	–0.097	0.672
Appraisal and recognition	B6	2.40	0.854	0.064	–0.627	0.500
(α_tot_ = 0.87)	B41	2.37	0.772	–0.053	–0.461	0.738
	B43	2.39	0.778	–0.123	–0.501	0.713
	B44	2.37	0.852	–0.004	–0.675	0.674
	B47	2.26	0.720	0.168	–0.174	0.694
	B49	2.54	0.759	–0.230	–0.301	0.681
Curriculum coordination	B7	2.75	0.773	–0.357	–0.114	0.640
(α_tot_ = 0.80)	B9	2.97	0.697	–0.490	0.517	0.655
	B22	2.68	0.658	–0.451	0.264	0.551
	B39	3.10	0.642	–0.317	0.267	0.553
Effective Discipline Policy	B2	2.86	0.648	–0.416	0.606	0.460
(α_tot_ = 0.73)	B11	2.86	0.772	–0.247	–0.354	0.561
	B13	2.80	0.737	–0.330	–0.008	0.620
	B56	2.98	0.742	–0.455	0.093	0.432
Excessive work demands	B8	2.65	0.950	–0.187	–0.877	0.533
(α_tot_ = 0.76)	B14	3.07	0.769	–0.353	–0.603	0.635
	B17	2.62	0.883	–0.162	–0.675	0.449
	B21	2.99	0.835	–0.380	–0.632	0.621
Goal Congruence	B15	3.01	0.652	–0.450	0.752	0.590
(α_tot_ = 0.80)	B18	2.76	0.731	–0.402	0.115	0.521
	B19	3.04	0.682	–0.386	0.228	0.649
	B20	2.90	0.662	–0.302	0.287	0.672
	B45	2.92	0.685	–0.602	0.880	0.460
Professional Growth	B16	2.24	0.830	0.113	–0.651	0.530
(α_tot_ = 0.75)	B23	2.57	0.837	–0.043	–0.569	0.569
	B25	2.31	0.965	0.108	–1.00	0.398
	B33	3.02	0.690	–0.533	0.654	0.580
	B42	2.61	0.830	–0.175	–0.494	0.568
Participative Decision Making	B27	2.34	0.779	–0.021	–0.496	0.505
(α_tot_ = 0.74)	B32	2.54	0.690	–0.178	–0.176	0.576
	B46	2.66	0.717	–0.483	0.121	0.595
	B51	2.81	0.788	–0.438	–0.057	0.488
Professional Interaction	B28	2.71	0.724	–0.279	–0.051	0.592
(α_tot_ = 0.85)	B29	3.25	0.709	–0.744	0.495	0.674
	B31	2.94	0.711	–0.292	–0.070	0.587
	B36	2.78	0.637	–0.250	0.191	0.550
	B50	3.23	0.722	–0.772	0.611	0.585
	B53	3.35	0.671	–0.860	0.824	0.657
	B55	3.14	0.618	–0.224	0.090	0.576
Role Clarity	B48	2.67	0.814	–0.325	–0.322	0.288
(α_tot_ = 0.63)	B52	3.58	0.565	–0.954	–0.095	0.477
	B54	3.23	0.612	–0.376	0.415	0.478
	B57	3.02	0.641	–0.367	0.592	0.471
Student Orientation	B24	3.14	0.588	–0.261	0.742	0.524
(α_tot_ = 0.70)	B26	3.29	0.609	–0.314	–0.261	0.473
	B35	2.99	0.774	–0.689	0.478	0.418
	B38	3.17	0.649	–0.403	0.265	0.581
Supportive Leadership	B10	2.95	0.824	–0.483	–0.261	0.729
(α_tot_ = 0.91)	B30	2.81	0.836	–0.454	–0.238	0.852
	B34	2.93	0.816	–0.543	–0.058	0.831
	B37	2.83	0.710	–0.336	0.119	0.790
	B40	2.69	0.875	–0.391	–0.475	0.739

The subscales homogeneity was adequate: Cronbach’s alpha (α) coefficients ranged from 0.7 to 0.91, with the exception of Role Clarity (RC). The items in this sub-dimension resulted in being less coherent because of item 48, which refers to others’ expectations and, in fact, after its deletion, the α coefficient was 0.70, and it became acceptable. In the RC subscale, this was the only item that referred to others, and so its content was quite different and specific (item–total correlation 0.288).

Given its conservative nature, at first this item was included in the CFA, but its performance was quite scarce: a loading of 0.36 and an R-squared of 0.13. Consequently, item 48 was omitted in order to improve the measurement of the “role clarity” factor.

**FIGURE 1:**
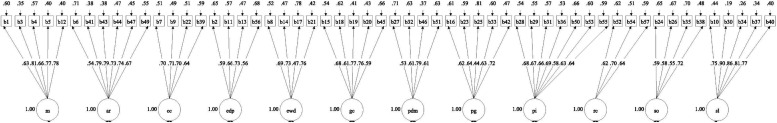
Factorial structure of the SOHQ.

A model based on the restricted set of 56 items resulted adequate, with all fit indexes being at least satisfactory (RMSEA = 0.07, SRMR = 0.05, CFI = 0.90), supporting the adequacy of the 12-factor structure for the SOHQ in the Italian sample. All parameters estimated in the confirmatory analyses were high (loadings from 0.40 to 0.89) and significant at the 0.001 level (Figure 1).

The estimated correlations showed that there was no significant relationship between Excessive Work Demands and Curriculum Coordination (*r* = 0.09, *p* > 0.68) and RC (*r* = 0.02, *p* > 0.77). Excessive Work Demands was significantly correlated with the other nine factors (*p* < 0.05), but the correlations, ranging between 0.12 and 0.37, were relatively small compared to other correlations between sub-dimensions. The correlations among the other 11 factors were in fact all significant at the 0.001 level and ranged between 0.28 and 0.84 (Table 2). Focusing on the Morale sub-dimension, it was strongly correlated with Effective Discipline Policy (*r* = 0.81), Goal Congruence (*r* = 0.78), Participative Decision Making (*r* = 0.76), and Professional Interaction (*r* = 0.77), and, although statistically significant, the correlation was weaker with Professional Growth (*r* = 0.28) and RC (*r* = 0.28).

**TABLE 2:** Correlations between SOHQ subdimensions.

	**1.**	**2.**	**3.**	**4.**	**5.**	**6.**	**7.**	**8.**	**9.**	**10.**	**11.**	**12.**	**13.**
1. M	–												
2. AR	0.60	–											
3. CC	0.63	0.54	–										
4. EDP	0.81	0.64	0.78	–									
5. EWD	0.28	0.23	*n.s.*	0.16	–								
6. GC	0.78	0.63	0.69	0.86	0.19	–							
7. PDM	0.76	0.76	0.59	0.83	0.31	0.83	–						
8. PI	0.77	0.63	0.76	0.86	0.25	0.76	0.80	–					
9. SO	0.52	0.45	0.54	0.69	0.12	0.78	0.62	0.70	–				
10. SL	0.55	0.60	0.43	0.67	0.36	0.62	0.85	0.57	0.57	–			
11. PG	0.67	0.84	0.53	0.72	0.37	0.81	0.87	0.73	0.65	0.74	–		
12. RC	0.28	0.45	0.51	0.63	*n.s.*	0.61	0.49	0.64	0.64	0.32	0.51	1	
13. JS	0.45	0.38	0.31	0.38	–0.26	0.39	0.32	0.35	0.24	0.33	0.36	0.30	1
14. LS	0.15	0.14	0.12	0.11	–0.12	0.11	*n.s.*	0.15	0.13	*n.s.*	*n.s.*	0.20	0.49

*All correlation significant, *p* < 0.01, with the exceptions of those indicated *n.s*. – not significant. M – morale; AR – appraisal and recognition; CC – curriculum coordination; EDP – effective discipline policy; EWD – excessive work demands; GC – goal congruence; PDM – participative decision making; PI – professional interaction; SO – student orientation; SL – supportive leadership; RC – role clarity; JS – job satisfaction; LS – life satisfaction.*

Finally, all the SOHQ sub-dimensions significantly correlated with job satisfaction at the 0.001 level, with *r* values ranging between 0.24 and 0.45. On the other hand, only four SOHQ sub-dimensions correlated with general life satisfaction at the 0.001 level (i.e., Morale, Appraisal and Recognition, Professional Interaction, and Role Conflict), whereas Curriculum Coordination, Effective Discipline Policy, Student Orientation, and Excessive Work Demands correlated at the 0.05 level with general life satisfaction; the remaining sub-dimensions reported non-significant associations.

## Discussion

The aim of the present study was to adapt the SOHQ to an Italian school context. CFA provided evidence for the adequacy of psychometric properties, highlighting that the SOHQ represents a valid tool for monitoring staff perceptions of the school climate and their morale. Therefore, these findings confirmed those of previous healthy promoting school (HPS) surveys, which have adopted the SOHQ with efficacy (Stewart et al., 2004).

Nowadays, investing in positive school climates is a key issue in schools, for the reciprocity of teacher and student wellbeing. The HPS “ethos” (Penney et al., 2017) highlights the positive gain circle where healthy school environments improve students’ growth and resilience, and, therefore, resilient students may strengthen future communities.

In the Italian context, where the present study has been conducted, schools are subject to different standardized processes of external evaluation [such as the PISA (Program for International Student Assessment) and the systematic assessment performed by the INVALSI (Istituto Nazionale per la Valutazione del Sistema di Istruzione e Formazione/National Institute for the Evaluation of the Education and Training Education System)] that are intended to assess, on one hand, students’ learning outcomes, and, on the other one, the quality of process outcomes, such as ability in planning training courses and a learning environment with inclusion and differentiation criteria of students’ needs. Despite these aims, the evaluation is only focused on students’ learning outcomes, but it could be helpful to analyze, along with these, the systemic conditions that represent the general frame where those outcomes are developed, like organizational and managing practices, leading processes, strategic orientation and the organization of the school in the identification of the objectives and their monitoring, human resource development and valorization (Aldridge and Fraser, 2018), and the integration with the social context, with the same perspective as HPS projects. In this vein, the SOHQ represents a valid tool for schools looking to evaluate several aspects of the organizational climate and consider teachers’ wellbeing at the same time. Results from this study have shown that all the organizational climate factors significantly correlate with morale, evidencing moreover a strong correlation with the aspects of organizational behavior and human resource management (e.g., professional growth, goal congruence, and participative decision making) which, differently from other questionnaires, have been specifically assessed by the SOHQ. Moreover, it has been evidenced that significant correlations emerged between SOHQ sub-dimensions and job satisfaction. Therefore, it can be stated that all the aspects of the school environment evaluated using this questionnaire represent relevant factors that are able to intercept the quality of working life and the wellbeing of teachers, thus representing a valid tool for school leaders in managing teachers’ work and planning school courses.

Although this study also gives an important insight into the usefulness of this measurement tool for the Italian context, the sample is not representative of the entire Italian teaching population. Future studies could overcome this limit by using this tool on a larger sample that is representative of the larger Italian school system, widening its scope, for example, to the other two teaching levels – middle and high school – and comparing public and private school teacher perceptions of organizational climate.

Moreover, the SOHQ should be adapted to other non-English speaking countries in order to evaluate its psychometric characteristics and to improve cross-cultural comparisons.

## Data Availability

The datasets generated for this study are available on request to the corresponding author. The School Organizational Health Questionnaire is subject to copyright and cannot be used without the permission of Insight SRC Pty Ltd (info@insightsrc.com.au).

## Author Contributions

DC, BL, GG, GM, and SV contributed to research conception and design, and acquired the data. DC, MC, and BL involved in critical revision of the article content. BL, GM, and SV performed quant data analysis. All authors interpreted the data and drafted the manuscript.

## Conflict of Interest Statement

The authors declare that the research was conducted in the absence of any commercial or financial relationships that could be construed as a potential conflict of interest. The handling Editor declared a past collaboration with one of the authors MC.
