# Corrigendum: Organizational Climate and Teachers’ Morale: Developing a Specific Tool for the School Context – A Research Project in Italy

**DOI:** 10.3389/fpsyg.2019.02464

**Published:** 2019-11-01

**Authors:** Daniela Converso, Michela Cortini, Gloria Guidetti, Giorgia Molinengo, Ilaria Sottimano, Sara Viotti, Barbara Loera

**Affiliations:** ^1^Department of Psychology, University of Turin, Turin, Italy; ^2^Department of Psychological, Humanistic and Territorial Sciences, D'Annunzio University of Chieti–Pescara, Chieti, Italy

**Keywords:** school climate, teachers' morale, teachers' wellbeing, confirmatory factor analysis, adaptation, validation

In the original article, there was a mistake in Table 1 as published. We erroneously inserted the description of each item. The corrected Table 1 appears below.

**Table 1:** Items distribution: descriptive, and reliability analysis by subdimension.

	**Code**	**M**	**Sd**	**S**	**K**	**Corr. item-total**
Morale (α_tot_ = 0.85)	B1	2.99	0.683	−0.499	0.636	0.560
	B3	2.97	0.702	−0.436	0.331	0.754
	B4	2.70	0.724	−0.286	−0.043	0.606
	B5	2.78	0.786	−0.331	−0.205	0.731
	B12	2.59	0.731	−0.398	−0.097	0.672
Appraisal and recognition	B6	2.40	0.854	0.064	−0.627	0.500
(α_tot_ = 0.87)	B41	2.37	0.772	−0.053	−0.461	0.738
	B43	2.39	0.778	−0.123	−0.501	0.713
	B44	2.37	0.852	−0.004	−0.675	0.674
	B47	2.26	0.720	0.168	−0.174	0.694
	B49	2.54	0.759	−0.230	−0.301	0.681
Curriculum coordination	B7	2.75	0.773	−0.357	−0.114	0.640
(α_tot_ = 0.80)	B9	2.97	0.697	−0.490	0.517	0.655
	B22	2.68	0.658	−0.451	0.264	0.551
	B39	3.10	0.642	−0.317	0.267	0.553
Effective Discipline Policy	B2	2.86	0.648	−0.416	0.606	0.460
(α_tot_ = 0.73)	B11	2.86	0.772	−0.247	−0.354	0.561
	B13	2.80	0.737	−0.330	−0.008	0.620
	B56	2.98	0.742	−0.455	0.093	0.432
Excessive work demands	B8	2.65	0.950	−0.187	−0.877	0.533
(α_tot_ = 0.76)	B14	3.07	0.769	−0.353	−0.603	0.635
	B17	2.62	0.883	−0.162	−0.675	0.449
	B21	2.99	0.835	−0.380	−0.632	0.621
Goal Congruence	B15	3.01	0.652	−0.450	0.752	0.590
(α_tot_ = 0.80)	B18	2.76	0.731	−0.402	0.115	0.521
	B19	3.04	0.682	−0.386	0.228	0.649
	B20	2.90	0.662	−0.302	0.287	0.672
	B45	2.92	0.685	−0.602	0.880	0.460
Professional Growth	B16	2.24	0.830	0.113	−0.651	0.530
(α_tot_ = 0.75)	B23	2.57	0.837	−0.043	−0.569	0.569
	B25	2.31	0.965	0.108	−1.00	0.398
	B33	3.02	0.690	−0.533	0.654	0.580
	B42	2.61	0.830	−0.175	−0.494	0.568
Participant Decision Making	B27	2.34	0.779	−0.021	−0.496	0.505
(α_tot_ = 0.74)	B32	2.54	0.690	−0.178	−0.176	0.576
	B46	2.66	0.717	−0.483	0.121	0.595
	B51	2.81	0.788	−0.438	−0.057	0.488
Professional Interaction	B28	2.71	0.724	−0.279	−0.051	0.592
(α_tot_ = 0.85)	B29	3.25	0.709	−0.744	0.495	0.674
	B31	2.94	0.711	−0.292	−0.070	0.587
	B36	2.78	0.637	−0.250	0.191	0.550
	B50	3.23	0.722	−0.772	0.611	0.585
	B53	3.35	0.671	−0.860	0.824	0.657
	B55	3.14	0.618	−0.224	0.090	0.576
Role Clarity	B48	2.67	0.814	−0.325	−0.322	0.288
(α_tot_ = 0.63)	B52	3.58	0.565	−0.954	−0.095	0.477
	B54	3.23	0.612	−0.376	0.415	0.478
	B57	3.02	0.641	−0.367	0.592	0.471
Student Orientation	B24	3.14	0.588	−0.261	0.742	0.524
(α_tot_ = 0.70)	B26	3.29	0.609	−0.314	−0.261	0.473
	B35	2.99	0.774	−0.689	0.478	0.418
	B38	3.17	0.649	−0.403	0.265	0.581
Supportive Leadership	B10	2.95	0.824	−0.483	−0.261	0.729
(α_tot_ = 0.91)	B30	2.81	0.836	−0.454	−0.238	0.852
	B34	2.93	0.816	−0.543	−0.058	0.831
	B37	2.83	0.710	−0.336	0.119	0.790
	B40	2.69	0.875	−0.391	−0.475	0.739

Furthermore, in the original article, there was an error. We neglected to include a copyright clause regarding the “School Organizational Health Questionnaire”, around which this article was based on.

A correction has been made to the **Data Availability** statement:

“The School Organizational Health Questionnaire is subject to copyright and cannot be used without the permission of Insight SRC Pty Ltd (info@insightsrc.com.au).”

The authors apologize for these errors and state that this does not change the scientific conclusions of the article in any way. The original article has been updated.

